# Repair of Double Orifice Left AV Valve (DOLAVV) with Endocardial Cushion Defect in Adult

**DOI:** 10.21470/1678-9741-2016-0034

**Published:** 2017

**Authors:** Vivek Velayudhan Pillai, Jayakumar Karunakaran

**Affiliations:** 1 Sree Chitra Tirunal Institute for Medical Sciences and Technology, Department of Cardiovascular and Thoracic Surgery, Trivandrum, Kerala, India.

**Keywords:** Mitral Valve Insufficiency, Endocardial Cushion Defects, Mitral Valve Annuloplasty

## Abstract

Double orifice left atrioventricular valve (DOLAVV) or double orifice mitral valve (DOMV) is a rare congenital cardiac anomaly manifesting either as an isolated lesion (mitral stenosis or mitral insufficiency) or in association with other congenital cardiac defects. Signs of mitral valve disease are usually present along with the symptoms of associated coexistent congenital heart diseases. Mitral insufficiency due to annular dilatation is seen when DOLAVV is associated with endocardial cushion defects. Surgical intervention like mitral valve repair or replacement is required in 50% of patients and yields good results. We report a case of a 56-year-old lady who successfully underwent surgical correction of DOLAVV with partial atrioventricular canal defect.

**Table:** 

Abbreviations, acronyms & symbols	
DOLAVV	= Double orifice left atrioventricular valve
DOM	= Double orifice mitral valve
VNYHA	= New York Heart Association

## INTRODUCTION

Double orifice left atrioventricular valve (DOLAVV) or double outlet mitral valve (DOMV) is a rare congenital cardiac defect characterized by mitral valve with 2 orifices inside a common fibrous annulus opening into the left ventricle. About 200 cases have been reported in the literature^[1]^. DOLAVV is mostly associated with endocardial defects, ventricular septal defect, coarctation of the aorta, interrupted aortic arch, subaortic stenosis, patent arterial duct, *secundum* atrial septal defect, tetralogy of Fallot, hypoplastic left heart syndrome, Ebstein's anomaly or bicuspid aortic valve. Chordopapillary anomalies, such as double parachute mitral valve and mitral valve leaflet clefts, have been reported along with DOLAVV.

## CASE REPORT

Fifty-six-year-old female presented with New York Heart Association (NYHA) class II dyspnoea and pedal edema (feet swelling). Clinically, the patient had a 3/6 grade pansystolic murmur in mitral area, with normal S1S2 heart sounds. Electrocardiogram showing complete atrioventricular block pattern. Chest X-ray showed cardiomegaly with prominent pulmonary arteries. Echocardiogram showed a partial atrioventricular septal defect, *ostium primum* atrial septal defect, moderate mitral insufficiency with a mitral cleft valve with parachute appearance.

**Fig. 1:**
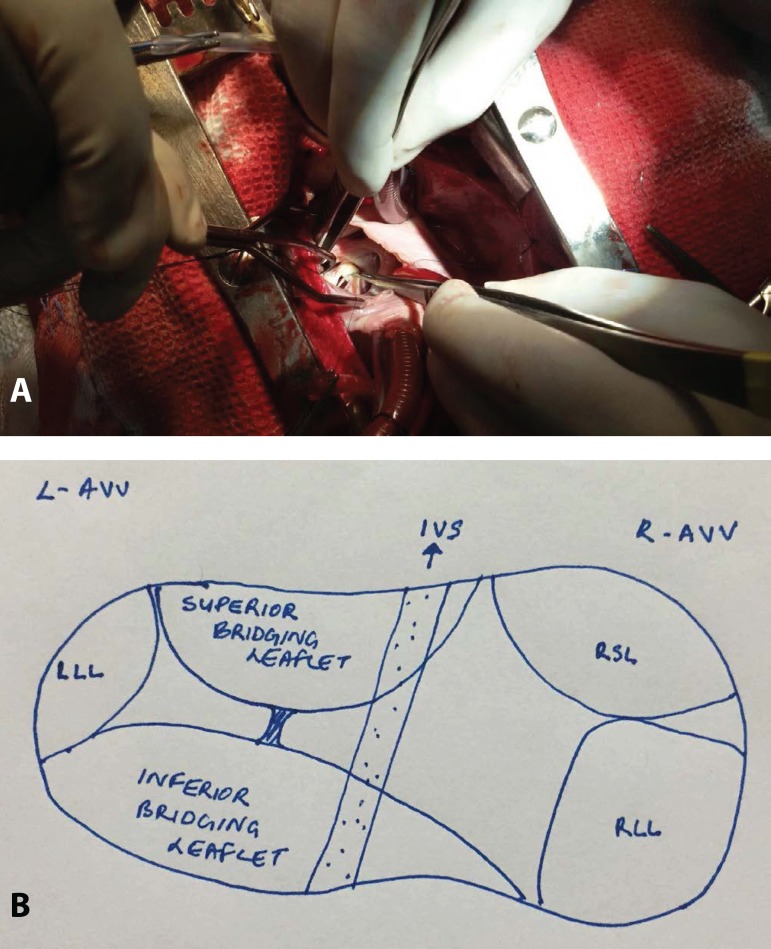
A: Intraoperative picture showing the two orifices of the mitral valve. B: Sketch of atrioventricular valves and left atrioventricular valve is a double orifice mitral valve.

**Fig. 2:**
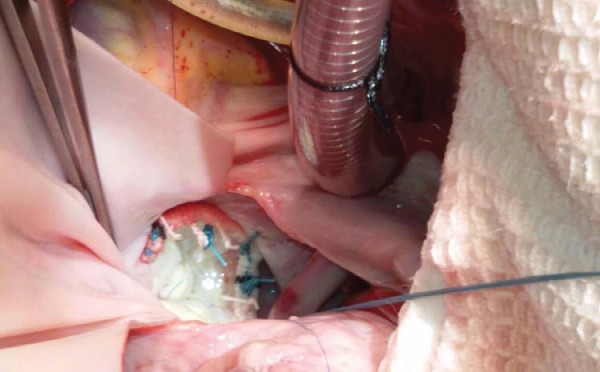
Competent mitral valve on saline testing after mitral annuloplasty.

Mitral valve was approached through the right atrium and *ostium primum* septal defect. Surgical findings include *secundum* atrial septal defect, partial atrioventricular septal defect, moderate mitral and tricuspid regurgitation. Left atrioventricular valve was of eccentric type double orifice mitral valve (Figure 1) guarding left ventricular inlet with two papillary muscles present as subvalvular apparatus supporting each orifices with chordae supporting the free edges and a dilated mitral annulus. The left atrioventricular valve (mitral valve) had good mobile leaflets with free edges supported by chordae. Gluteraldehyde fixed autologous pericardial patch closure of *primum* and *secundum* atrial septal defects was performed. Atrioventricular valve was bicuspidised by obliterating the posterior leaflet. Each mitral orifice was sized to the required Hegar size to rule out mitral stenosis. Since there was no chordal rupture, mitral insufficiency was found to be primarily due to annular dilatation and this was repaired by #31 Tailor (St. Jude Medical, MN, USA) ring mitral annuloplasty (Figure 2). Permanent pacemaker was placed since the patient was preoperatively in complete atrioventricular block. Follow-up echocardiography showed no residual atrial septal defects, mitral regurgitation of 1+ with mitral valve gradient of 3/1 mmHg and tricuspid regurgitation of 1+. The patient had an uneventful postoperative recovery.

## DISCUSSION

DOLAVV is thought to be the outcome of either fetal endocarditis or developmental anomaly due to the retention of a part of the common atrioventricular valve which is normally resorbed.

Clinically significant mitral stenosis or mitral regurgitation is seen in half the number of diagnosed patients. Congestive heart failure that occurs in endocardial cushion defect is accentuated by the abnormal mitral valve^[2]^. Pulmonary venous hypertension is the cause of symptoms like tachypnea, dyspnea and cough. Two-and-three-dimensional echocardiography delineates the anatomy of the mitral valve^[3,4]^.

Morphologically, the mitral valve has two openings into the left ventricle-anterolateral and posteromedial orifices. Physiologically significant mitral stenosis or mitral regurgitation are seen in 50% of cases. It is reported that 25% of patients with DOLAVV have endocardial cushion defect and around 5% of patients with endocardial cushion defect have DOLAVV. Anatomically there are three different variants of DOLAVV namely hole type (85%), complete bridging type (15%) and the incomplete bridging type. In the complete bridge type there is a central connecting leaflet tissue joining the two leaflets, dividing the mitral orifice into a medial and a lateral part^[5]^. The orifices may be equal or unequal^[3]^. When associated with an atrioventricular canal defect, the accessory orifice is located at the posteromedial commissure. The papillary muscles are usually well formed and normal, with supporting chordae surrounding each orifice and attaching into one adjacent papillary muscle.

Management depends on the type and severity of mitral valve dysfunction. Isolated DOLAVV causing neither obstruction nor regurgitation needs no active intervention. Mitral valve repair (neochordae and annuloplasty), cleft closure and mitral valve replacement yields good surgical result.

## CONCLUSION

Surgical intervention is necessary when mitral stenosis or incompetence is severe or if repair of an associated cardiac lesion is needed^[6]^. Surgery is relatively safe and gives good results.

**Table:** 

Authors' roles & responsibilities	
VVP	Conception and study design; realization of the operation; analysis and/or data interpretation; manuscript redaction or critical review of its content; final manuscript approval
JK	Conception and study design; realization of the operation; analysis and/or data interpretation; manuscript redaction or critical review of its content; final manuscript approval
